# Self-Renewal of Stem Cells

**Published:** 2009-07

**Authors:** V.V. Terskikh, Ye.A. Vorotelyak, A.V. Vasiliev

**Affiliations:** 1 N.K. Koltsov Institute of Developmental Biology, Russian Academy of Sciences

## Abstract

Asymmetric division is one of the most fundamental characteristics of adult stem cells , which ensures one daughter cell maintains stem cell status and the other daughter cell becomes committed to differentiation. New data emerged recently that allow us to conclude that asymmetric division has another important aspect: it enables self-maintenance of stem cells.

## INTRODUCTION

The central aspect of stem cell biology is asymmetric division. Earlier it was suggested that with the help of asymmetric division two problems could be solved at the same time: one daughter cell preserves the qualities of the stem cell and continues to self- renew, whereas the other acquires the ability to differentiate [1. 2, 3, 4]. Stem cell niches create an asymmetric microenvironment and control local processes of proliferation and differentiation of stem cells through the integration of signals from neighbor cells, from the organism, and from the external environment [5]. Niches create a system of signals directed toward the maintenance of stem cells. That has been studied in detail on germline stem cells in Drosophila. For example, it was shown on the germinal stem cells in the Drosophila ovary how the signal from the stromal cells (Dpp) regulates the self-renewal of the stem cells and influences the fate of the daughter cells [6]. In the process of ontogenesis and during the neoplastic transformation, stem cells may divide both symmetrically and asymmetrically, depending on the circumstances under which they reside [2].

Asymmetric division and cell-cell interactions are universal mechanisms of the formation of cell diversity and are of primary importance in development of multicellular organisms. Diversity of cell types may be created in two major ways [7]. One way is when a great number of identical cells are initially formed which later acquire various ways of differentiation due to cell-cell interaction. In another case, daughter cells become different from their time of birth when, in the process of mitosis of polarized mother cell, cell fate determinants segregate only in one of the daughter cells. This distribution of determinants provides for specialization of one daughter cell in a certain way, which differs from the specialization of the sister cell.

In order for the asymmetric division to proceed successfully, it is necessary that several key processes take place: (1) the cell undergoing the division should be initially polarized. Polarization may include differences in the structure of certain parts of the cell membrane and uneven distribution of determinants in the cortex and in the cytoplasm of the cell. (2) Mitotic spindle is oriented parallel to the cell polarization axis. (3) The forming mitotic spindle is also asymmetric. This results in the fact that the two centrosomes that form the spindle are different (4). As a result of the division, the cell fate determinants are distributed asymmetrically between the daughter cells.

Both ways of cell differentiation and the strategy of development can be seen in closely related nematodes [8]. Early development starts from asymmetric mitoses in Caenorhabditis elegans and Acrobeloides nanus, and the formed cells have strictly determined fate: out of the 949 mitoses that appear during C. elegans development, 807 are asymmetric. In Enoplus brevis nematode, identical blastomeres are formed first which differentiate during the further development as a result of asymmetric divisions. Asymmetric division is a conservative mechanism that provides the possibility of daughter cells development in different directions, which is why the problem of asymmetric division is of fundamental importance to developmental biology and, in particular, to the biology of stem cells [9, 7, 10]. Asymmetric division has been identified in different groups of organisms: in bacteria [11, 12], yeasts [7], Volvox [13], nematodes [14], Drosophila [15], vertebrates [16, 17], and plants. Several subjects have been thoroughly studied and are considered classical: dividing Drosophila neuroblasts [15] and division of first blastomeres in Caenorhabditis elegans [14, 18]. A prerequisite for asymmetric cell division apparently exists in all organisms, but whether or not it happens depends on the particular situation. It is possible to state now that two programs are written in a cell: for symmetric and asymmetric division. In development and during tumor transformation, stem cells may divide both symmetrically and asymmetrically, depending on their microenvironment [2]. For example, during the embryonic development of mouse, asymmetric division is directed toward regulating the number of neural stem cells. During the 12-16 d of gestation in the ventricular brain zone, a lot of apoptotic cells are found and, at the same time, ceramide expression increases. It was shown [19] that, at that time, the neural progenitor cells divide in such a way that asymmetric distribution of the nestin and prostate apoptosis response 4 (PAR-4) occurs. As a result of such division, one daughter cell is PAR-4+ nestin-, in which the increased contents of ceramide incite apoptosis, and another cell not expressing PAR-4 is nestin-positive and not subjected to apoptosis. Besides, the asymmetric division is functionally connected with the apoptosis, because some genes involved in the regulation of the asymmetric division control signal pathways responsible for apoptosis [20]. Transition of daughter cells to apoptosis, as well as to the differentiated state, may be used in the organism for maintaining the cell homeostasis. In Drosophila, mutations in some genes in the homozygous state cause the disturbance of the apical-basal cell polarity and disorganization of the epithelium structure; this is why these genes were termed cell polarity genes . Formation and maintenance of the apical-basal cell polarity is of great importance for their functioning and for undergoing asymmetric mitosis. Polarization of cells is controlled by the complex interaction of a great number of genes [21, 22]. The loss of cell polarity and the succeeded alteration of asymmetric mitosis may result in the loss of proliferation control and that, in turn, may induce the chain of events leading to the malignant growth. It was shown in Drosophila neuroblasts that genes controlling asymmetric mitosis may be tumor suppressors, and mutations of those genes induce neoplastic growth [23, 24].

## The asymmetric division of Drosophila neuroblasts

One of the most studied models of asymmetric division is neural progenitor cells (neuroblasts) of Drosophila that produce the majority of the central nervous system cells. Neuroblast undergoes asymmetric division and produces two daughter cells of different sizes. The bigger cell maintains the qualities of the neuroblast and may divide several times asymmetrically, whereas the smaller daughter cell, which is called the mother ganglion cell, is committed to differentiation and divides only once, producing two neurons or two glia cells. A large number of protein complexes take part in cell polarization [25, 26]. Apical-basal polarization of the neuroblast takes place in the late G2, when the set of proteins called Par complex localizes in the apical part of the cell. For the successful proceeding of mitosis, correct localization of the protein complexes in the apical cortex of neuroblast is needed [24]. Segregation of baso-lateral and apical protein complexes is based on their antagonism that brings about their polar distribution in the cell.

In Drosophila neuroblast, the apically localized proteins form two complexes interconnected by the adaptor Inscuteable protein. The evolutionally conserved Par complex includes Bazooka/Par3, aPKC, and Par6 and is the first complex of proteins that localizes in the cell cortex of the neuroblast and is initially involved in the displacement of the proteins from the apical cortex, the proteins that localize in the basal part of the cell. This protein complex regulates the activity of the tumor suppressor Lgl (lethal giant larvae), which is also necessary for the correct targeting of basal protein complexes. Lgl is directly associated with Par6 and, in this complex, it seems that aPKC inactivates Lgl through its phosphorylation. Due to the activity of the non-phosphorylated Lgl, Miranda protein is recruited in the basal cortex.

The second apical protein complex contains proteins bound to the signaling way of the heterotrimeric G-protein and includes Gαi, Partner of Inscuteable (Pins), and Locomotion defects (Loco). The Gαi-Pins-Loco complex intercedes the mitotic spindle formation, as well as its correct position (in a way parallel to the apical basal axis), toward the plane of the neuroblast division.

The mitotic spindle of the neuroblast is asymmetric, its length is longer in the apical part, and, as a result, it is shifted to the side of the basal cortex. That is why, as was mentioned earlier, two cells of different sizes are formed. Centrosomes in the Drosophila neuroblast, which are under division, appear to be non-equivalent; the mother centrosome, which is bigger, is surrounded by more extensive astral microtubules and remains in the neuroblast in later divisions.

Due to tumor suppressors Discs large(Dlg) and Lethal (2) giant larvae (Lgl) localized in the cortex, the apical Par complex provides the basal localization of the RN A-binding Staufen protein, the transcription Prospero (Pros) factor, the Numb protein which associates with plasma membrane, and the adaptor proteins Miranda (Mira) and Partner of Numb (Pon) [27, 28]. Tumor suppressor Lgl, which is the cytoskeletal protein and which directly binds with non muscular myosin II (Zipper), suppresses its activity and prevents binding with the apical complex. Lgl is evenly distributed through the entire cell cortex. However, in the area of the apical cortex, the aPKC phosphorylates and inactivates Lgl, releasing myosin II. Activated myosin II may form filaments and displace the Miranda protein. On the contrary, Lgl is active in the basal cortex because of aPKС absence and it suppresses the activity of myosin II, which allows the Miranda to locate in the basal cortex [29]. In contrast to myosin II, which displaces the determinants from the apical cortex, myosin VI (Jaguar) provides basal localization and segregation of Mira/Pros by means of vesicular transport [30].

The Pins protein may associate with the protein Mud (mushroom body defective) of the mitotic apparatus, which is associated with the centrosome and apical cortex that is necessary for the correct orientation of the spindle. Dlg and the Khc-73 (Kinesin-73) protein situated on the plus ends of the astral microtubules are also necessary for correct positioning of the spindle. Actinomyosin cytoskeleton plays an important part in the assembling of these apical and basal protein complexes. It seems that actin filaments, not the microtubules, take part in the binding of proteins with the cortex. Drosophila myosins II and VI are present in mutually exclusive complexes with Miranda and are necessary for the correct localization of the determinants that determine the fate of the cell. Asymmetric Numb localization is regulated by the phosphorylation cascade that triggers the activated Aurora-A. This kinase phosphorylates Par-6, the regulatory aPKC subunit, which triggers the activation of aPKС. This in turn leads to the phosphorylation of Lgl, which binds and suppresses aPKC activity in the interphase. Phosphorylated Lgl becomes free from aPKC and allows for Bazooka to take its place in the protein complex. As a result, the specificity of the substrate changes and aPKC is able to phosphorylate Numb. Phosphorylated Numb is localized asymmetrically as a crescent in the basal part of the cell [31]. Proteins of the basal part of the neuroblast form two complexes. One of these complexes contains Miranda adaptor protein that is associated with transcription repressor Brat (Brain tumor) and assists in its asymmetric localization, the homeodomain transcription factor Prospero, and the protein Staufen, which binds the two-stranded RN A and which can itself bind prospero transcripts. The second complex contains Numb, the Notch protein antagonist, and binding it protein Pon (Partner of Numb). After segregation into the mother ganglion cell, Miranda degrades, which allows Prospero translocation into the nucleus and activation of genes involved in the differentiation and repression of genes involved in proliferation processes. Mitotic spindle plays an active part in the process of asymmetric division. It has been shown on several objects that it is created by structurally and functionally different centrosomes. Mitotic spindle also appears to be asymmetric, because it is formed by structurally and functionally different centrosomes. For the yeasts S. cerevisiae [32, 33], "c o m p a s model" was proposed which suggests that mitotic spindle, like the magnetic needle of a compass, localizes in the cell not passively but reacts to the signals from the cortical layer of the cytoplasm. During daughter cell budding, the Kar9 protein, which is necessary for correct spindle orientation, is located at the pole that is oriented to the side of the daughter cell. Then, Kar9 moves from the pole to the microtubules, which are directed to the specific parts of the cortical layer of cytoplasm in the daughter cell. This type of model suggests that spindle asymmetry is necessary for reaction to the cortex signals and for correct orientation in a dividing cell. In Drosophila neuroblasts [34] and in embryonic cortex of murine brain [35], the asymmetric cell division is accompanied by the active movement of mitotic spindle. However, in Drosophila germinal stem cells, the centrosomes take their final place in the interphase, and asymmetric division proceed with the permanent spindle position [36].

## The asymmetric division of higher organisms

Asymmetric division in higher organisms has yet to be studied sufficiently. Sporadic findings show that such divisions take place. In many epithelial tissues, both symmetric and asymmetric cell divisions are detected. For example, during symmetric mitoses, both cells are morphologically identical and situated on the basal membrane; during asymmetric mitosis, the daughter cells are morphologically different, whereas one of them transits immediately to the epithelial suprabasal layer. It is possible to suggest that during the asymmetric and symmetric divisions different mechanisms of cell migration to the suprabasal layer can exist. In the basal cells of the human esophagus, epithelium asymmetric division has been described [37], during which mitotic spindle is oriented perpendicularly to the basal membrane, which is why one daughter cell retains contact with the basal membrane and another moves into the suprabasal layer. The authors suggest that this is how stem cells divide. In a mouse epidermis on the 12.5 d of embryonic development, the large part of epidermis consists of one layer and the overwhelming number of cell divisions take place in the epithelium plane (i.e. they are symmetric); however, some cells divide perpendicularly to the basal membrane. While multilayered epithelium appears after 15.5 d of gestation, more than 70% of the cells have vertically aligned spindle. Evidently, stratification of epidermis resulted from asymmetric mitotes [38]. In mouse tail epidermis, about 30% of the basal layer cells may undergo asymmetric division [39]. Lamprecht [40] showed that, in the basal cells of rat cornea epithelium, both symmetric and asymmetric mitoses occur.

Single progenitor haematopoietic cells isolated from the human fetal liver undergo asymmetric divisions in vitro [41]. It was found that approximately 30% of CD34+ cells gave birth to two daughter cells with different behaviors. One cell remained quiescent for 8 d, whereas the other started to proliferate exponentially with a doubling time of 12 h. Even more often ( circa 40% of cases) asymmetric division was found in CD34+ CD38- cells. Asymmetric division in mammalian stem cells still remains insufficiently studied, but some indirect findings suggest such a possibility. Mammalian tissues comprise small fractions of stem cells, about one percent or several percents, and, in many cases, they are very hard to identify in situ. The role of asymmetric segregation of the determinants in the cells of vertebrates has practically not been studied: however, homologues of some genes that provide the origin of Drosophila asymmetric mitosis were discovered. The evolutionally conservative numb gene was discovered in many vertebrates [44, 45]. Asymmetric divisions take place in the cells of the ferret cerebral cortex [42] and in the stem cells of the mouse cerebral cortex and neuroblasts [43]. And in all cases accomplishment of asymmetric division, just like in Drosophila neuroblasts, it is necessary to have an asymmetrically distributed Numb factor. Asymmetric Numb localization has been found in dividing satellite cells of mouse [46]. In Drosophila, Numb function is to suppress Notch signaling during neurogenesis. In vertebrates, Numb fulfills the same functions as in Drosophila neuroblasts and takes part in the regulation of the asymmetric division of mammalian cells [45, 47, 48]. Two Pins homologues [49] were found in vertebrates. In rats, the AGS-3 corresponds to the Pins protein, which is expressed only in some tissues. Another Pins homolog, LGN, is expressed in many human tissues. During the interphase, this protein is in the cytoplasm, and it is associated with the poles of the spindle during mitosis. Suppression of the LGN expression destroys the spindle organization and prevents chromosomes from normal disjunction [50]. Insc functioning is necessary for the correct orientation of asymmetric mitosis in the progenitor cells of rat retina [51]. Also, homologues for Par-3, Par-6, and aPKC have been found.

## Aging stem cells

The question of age-related changes in stem cells in the course of aging of the organism and its tissues where stem cells are localized is of crucial importance for stem cell biology [52]. In quickly renewing tissues (such as blood, epidermis, and the intestinal epithelium), stem cells make up a remarkable component and have a big proliferative potential. Mouse hematopoietic stem cells function throughout the lifetime of the animal, and serial transplantations have shown that the life expectancy of stem cells may significantly exceed the life expectancy of the organism. It was shown in many experiments that aging, as well as younger bone-marrow cells are able to restore hematopoiesis in recipients after repeated transplantations [53, 54, 55]. After several rounds of transplantation, the ability of stem cells to rescue lethally irradiated animals is lower: however, it is worth noticing that, at the same time, the proliferative potential of stem cells may not be exhausted, but unfavorable effects may be connected with the technique of isolation and transplantation of stem cells, as well as the radiation treatment of recipient niches [56, 57}. This makes it possible to suggest that, with the organism aging, no essential lowering of the proliferative potential occurs in stem cells. Naturally, then, the question arises as to whether stem cells age or not. This question cannot be unambiguously solved right now. Stem cells aging can be of a replicative character (as a result of the accumulation of errors during the repeated proliferative cycles) and of a chronological character, connected with different aspects of stem cell behavior. Though the hemopoietic stem cells (HSC) of younger and older mice were similar in their ability to restore hematopoiesis, older animals had five times more HSC than the younger ones: however, they were worse at finding niches and engrafting the bone marrow of irradiated recipirnts. HSCs of young animals were predominantly quiescent, whereas in older animals they were more often in the proliferative cycle [58, 59]. Clonal analysis of the repopulating HSCs showed that aging animals have a diminished number of lymphoid-biased HSCs, while the number of the long-term HSCs of the myeloid series rises. Myeloid HSCs of the younger and older animals behave in the same way in all aspects. This leads us to suggest that aging does not influence the qualities of individual SC, but it affects the clonal composition of the HSC. Evidently, the reduction in the level of lymphocytes in the blood may be an indicator of HSC aging [60]. Rossi et al. [61] showed that in aging animals endogenous DNA errors accumulate in stem cells; that may be the cause of cell aging and can be reflected in the SC functioning and maintenance of the tissue homeostasis during stress. Behavior of HSCs as the organism ages may also depend on the genetic factors manifested in different lines of mice. The number of HSCs in DBA line barely changes with the age of animals, and the number of young HSCs even falls, whereas both factors in older animals significantly increase in C57BL/6 line.

There are grounds to believe that age-related changes in stem cells are reversible, because in skeletal muscle satellite cells of mouse, it was shown [63] that a rejuvenation of the satellite cells of older animals takes place during heterochronic parabiosis. Age-related changes in the HSCs of mice may also be reversible [64]. As for germinal SC, significant aging of niches where they are located was demonstrated [65]. Embryonic SCs cultivated in vitro apparently do not age [66]. During the lifetime of a mouse, no pronounced aging or lowering of the physiological functions of epidermal SCs was discovered [67], which may be connected with the special biological significance of the barrier function of epidermis in the life of animals. These findings make it possible to speculate about age-specific reversible (epigenetic) changes in the SCs and about long-term retaining of their proliferative potential. On the whole, it is possible to conclude that the number of tissue SCs and their functioning may change as the organism ages. However, the stem cells retain their ability to self-renew. One of the processes connected with cell aging is the formation of intracellular protein inclusions. Correct folding of the nascent proteins in the cell requires the participation of different protein cofactors known as molecular chaperones. Those molecules recognize and bind growing chains of polypeptids and partly folded proteins in order to provide them with the native conformation and prevent misfolding and subsequent aggregation. There are several chaperone families, including heat shock proteins. Throughout the cell cycle, permanent synthesis and degradation of proteins take place. Misfolded proteins or the proteins damaged due to oxidative stress or heat shock are destroyed in the cell because of proteolysis; however, the cells appear to be unable to degrade the misfolded and damaged proteins in some situations [68], and they may form microaggregates. In higher eucariots, those microaggregates accumulate in the aggresomes which are formed as a result of direct transportation of microaggregates from the cell periphery to centrosomes or microtubules organization centers, where they are surrounded by intermediate microfilaments [69]. Formation of aggresomes is the generalized cell answer to the clustering of the aggregated nondegraded proteins. After inclusion in aggresomes, proteins are not able to undergo degradation by proteasomes. Aggregation of a large number of aggresomes ("biological garbage") is considered one of the important factors of cell aging and dying [70, 71]. Formation of aggresomes may be the reason for the dysfunction and death of postmitotic cells such as neurons and cardiomyocytes. Many neurodegenerative diseases, including Alzheimer's, Parkinson's, and Huntington's diseases, are characterized by the selective death of neurons due to aggresomes formation resulting from abnormal processing of the mutant, misfolded or damaged proteins by the ubiquitin- proteasome system [72]. For example, the mutant protein Huntingtin (Htt), which characterizes for Huntington's disease, contains a polyglutamine fragment which assists aggresome formation. Arrasate et al. [73] showed that, when there is an increase in the quantity of diffused Htt in cells, the death of particular neurons occurs. Microaggresomes accumulation, along with the formation of inclusion bodies, increases the vitality of neurons and protects them from the toxic effect of Htt. In a similar way, aging cells accumulate oxidized proteins, e.g., carbolinylated proteins which form high-molecular aggregates that are not subjected to degradation [74]. To a certain degree, aggresomes formation near centrosomes does not influence the correct organization of the spindle and mitosis flowing: however, when there is a great excess of aggresomes, mitosis and cell functions are disturbed [75]. The functional inequality of the centrosomes in the cell causes asymmetric orientation of the spindle. This was shown for Drosophila neuroblasts [76], germinal stem cells of Drosophila [77], and budding yeasts cells [32]. The centrosomes asymmetry is expressed particularly in that aggresomes are accumulated only around one of them [75]. Because the mechanism of asymmetric division in Drosophila neuroblasts is well studied and the neuroblasts themselves are frequently used for modeling the stem cells behavior, they were chosen for an examination of the mutant proteins behavior in the asymmetric mitosis [75]. A recombinant Drosophila was created in which the N-end fragment of the human Htt protein was expressed; this human protein contained 128 glutamine repeats (Htt-Q128). It was shown in the culture of the isolated neuroblasts that the aggregated Htt-Q128 protein usually formed a protein inclusion associated with only one pole of the spindle. As a result of the asymmetric division, the inclusion moved to the newly formed neuroblasts and mother ganglion cells were free from damaged proteins. These findings made it possible to suggest that the mechanism of aggresomes segregation in the process of symmetric mitosis may fulfill the same function in mammalian SCs. In Drosophila cells on the blastoderm stage, asymmetric divisions take place and, at the same time, the proteins predestined for degradation are distributed asymmetrically [78]. There is an indication that, in the stem cells of the crypt of small intestines of patients with type-3 spinocerebellar ataxy (ScA-3), an asymmetric distribution of the mutant protein ataxin-3 takes place [75]. This protein does not form inclusions in normal patients, but patients with ScA-3 manifest the aggresomes in committed and differentiated cells; however, they are not formed in the SCs situated at the crypt bottom near the Paneth cells. Judging by the microscopic inclusions that can be seen with an electron microscope, ataxin-3 is expressed in the crypt SCs as well; however, they are freed from the aggresomes after asymmetric mitosis. These findings make it possible to suggest that another exclusively important function of the asymmetric division is the self-renewal of the adult stem-cell line. In this case, one of the two daughter cells breaks free from the damaged non-degraded protein molecules and maintains its biological age, whereas the other daughter cell, which inherits the damaged molecules, either dies as a result of apoptosis or differentiates. Continuous proliferation is a necessary factor of self-renewal of adult SCs, because in nonproliferating cells damaged proteins accumulate and chronological aging of the cells takes place.
